# Avoidance of primary healthcare among transgender and non-binary people in Canada during the COVID-19 pandemic

**DOI:** 10.1016/j.pmedr.2022.101789

**Published:** 2022-04-04

**Authors:** Abigail Tami, Tatiana Ferguson, Greta R. Bauer, Ayden I. Scheim

**Affiliations:** a Department of Epidemiology and Biostatistics, Dornsife School of Public Health, Drexel University, Philadelphia, PA, USA; b Trans PULSE Canada, Toronto, Canada; c Department of Epidemiology and Biostatistics, Schulich School of Medicine & Dentistry, Western University, London, ON, Canada; d Li Ka Shing Knowledge Institute, St. Michael’s Hospital, Toronto, ON, Canada

**Keywords:** Transgender, Non-binary, Mental health, Healthcare avoidance, Primary care

## Abstract

Transgender (trans) and non-binary people experience barriers to culturally competent healthcare and many have reported avoiding care. COVID-19 and related mitigation strategies may have exacerbated avoidance, and poor mental health may be bidirectionally related to avoiding care. This study estimated the prevalence of primary care avoidance during the pandemic in a national sample of trans and non-binary people in Canada with a primary care provider and examined the association between poorer self-rated mental health and avoidance. In Fall 2019, Trans PULSE Canada collected multi-mode survey data from trans and non-binary people. In September to October 2020, 820 participants completed a COVID-19-focused survey. In this cross-sectional analysis, multivariable logistic regression models estimated odds ratios adjusted for confounders and weighted to the 2019 sample. The analysis included 689 individuals with a primary healthcare provider, of whom 61.2% (95% CI: 57.2, 65.2) reported fair or poor mental health and 25.7% (95% CI: 22.3, 29.2) reported care avoidance during the pandemic. The most common reason for avoidance was having a non-urgent health concern (72.7%, 95% CI: 65.9, 79.5). In adjusted analyses, those with fair or poor mental health had higher odds of avoiding primary care as compared to those with good to excellent mental health (adjusted odds ratio [AOR] = 2.37; 95% CI: 1.50, 3.77). This relationship was similar when excluding COVID-related reasons for avoidance (AOR = 2.52; 95% CI: 1.52, 4.17). Expansion of virtual communication may enhance primary care accessibility, and proactively assessing mental health symptoms may facilitate connections to gender-affirming mental health services.

## Highlights

• Transgender and non-binary populations experience barriers to healthcare.
• Poor mental health may contribute to healthcare avoidance.
• Existing disparities may have been worsened due to the COVID-19 pandemic.
• Trans PULSE Canada surveyed trans and non-binary individuals amidst COVID-19.
• Participants with worse mental health were more likely to avoid primary care during the pandemic.

## 1 Introduction

Transgender (trans) and non-binary populations, composed of individuals that have a gender identity that differs from their sex assigned at birth, face disparities in access to healthcare (Bradford et al., 2013). Socioeconomic factors, uninformed healthcare providers, and structural stigma in health institutions all limit access to healthcare for these populations (Safer et al., 2016, Gonzales and Henning-Smith, 2017, Grant et al., 2011, White Hughto et al., 2015). Further, due in part to enacted and anticipated discrimination, trans and non-binary people may avoid or postpone primary care visits (White Hughto et al., 2015, Cruz, 2014, Seelman et al., 2017, Kcomt et al., 2020). Access to care is particularly important for trans and non-binary populations, which experience a high prevalence of depression, anxiety, and substance misuse (Kcomt et al., 2020; Moagi et al., 2021, Newcomb et al., 2020, Su et al., 2016).

The novel coronavirus (COVID-19) pandemic and related emergency orders and mitigation efforts may have exacerbated mental health disparities and healthcare barriers established in trans and non-binary populations (Kidd et al., 2021, Alonzi et al., 2020, van der Miesen et al., 2020). For example, in a global survey of trans and non-binary individuals during the COVID-19 pandemic, anxiety and depression were both prevalent and directly linked to indicators of the pandemic environment (e.g., stay-at-home orders) (Restar et al., 2021). Socioeconomic loss and reduced access to gender-affirming services partially mediated the relationship between exposure to the pandemic and poorer mental health (Restar et al., 2021). These barriers may operate differently in the context of Canada’s universal public health insurance for primary care and relatively generous government financial relief for those who lost income as a result of COVID-19 (Government of Canada, 2021). Nonetheless, across 76 countries, many trans and non-binary individuals reported decreased access to gender-affirming support services or care during the COVID-19 pandemic (Jarrett et al., 2020). In the same global study, for those who experienced this reduced accessibility, the prevalence of mental health related symptoms was 1.61 to 1.74 times higher (Jarrett et al., 2020). In another international study assessing the impact of COVID-19 on healthcare access for trans individuals, investigators found that 49.3% of survey participants who had already undergone transition-related treatment reported restrictions in access to transgender-specific healthcare services (Koehler et al., 2021). Other COVID-19 mitigation efforts may also have impacted the mental health of trans and non-binary people in Canada. These include the closure of higher education institutions (Gonzales et al., 2020), social isolation measures (Salerno et al., 2020, Hawke et al., 2021), and consequently, reduced access to trans and non-binary community supports (Kidd et al., 2021, Trans PULSE Canada COVID Cohort Working Group on Behalf of the Trans PULSE Canada Team).

Mental health challenges, both pre-existing and exacerbated by the pandemic, can contribute to healthcare barriers and avoidance among trans and non-binary people (Safer et al., 2016, Wang et al., 2020). Avoidance of care may, in turn, worsen mental and physical health (Kcomt et al., 2020). Further, poor mental health may have made it particularly difficult for trans and non-binary people to navigate the healthcare system while service availability and delivery modes were restricted due to COVID-19-related emergency orders in Canada (Government of Canada Department of Justice, 2021). In an online survey of 342 trans and non-binary individuals in the United States during the pandemic, a majority of participants met symptom thresholds for depression and anxiety, and 16.1% had avoided needed healthcare since the beginning of the pandemic due to anticipated discrimination (Mason, 2021). However, the study did not examine the relationship between mental health status and healthcare avoidance.

This cross-sectional study aims to examine the relationship between self-rated mental health and primary care avoidance among trans and non-binary people living in Canada amidst the COVID-19 pandemic. Just prior to the pandemic, an estimated 81.4% of trans and non-binary people in Canada had a primary care provider (Scheim et al., 2021). We hypothesized that within this group, those with fair or poor self-rated mental health would be more likely to avoid primary care. By identifying potential gaps in care for trans people experiencing mental health challenges, results of this study may be used by healthcare providers and advocates to improve accessibility and approachability of care for trans and non-binary patients during the pandemic and beyond.

## 2 Methods

### 2.1 Data source and participants

Data for this analysis come from the Trans PULSE Canada COVID Study, an online survey in which data were collected via REDCap (Harris et al., 2009) in English and French, between September 21 and October 20, 2020. To be eligible, individuals needed to have completed the 2019 Trans PULSE Canada survey, have consented to recontact for additional research, and be currently residing in Canada. Trans PULSE Canada was a 2019 national, community-based research study on trans and non-binary health that used a multi-mode convenience sampling approach (Scheim et al., 2021). Eligible participants were Canadian residents aged 14 years and older in 2019, whose gender identity differed from their sex assigned at birth. The survey was promoted online (mailing lists, social media), in-person at lesbian, gay, bisexual, and transgender (LGBT) community groups and events (e.g., Pride festivals), and through outreach by Peer Research Associates (PRAs) in ten urban centers. The survey could be completed in English or French online, on paper (mailed with a self-addressed, stamped return envelope), by telephone (with or without a language interpreter), or on an electronic tablet with one of 11 PRAs. After completing the 2019 survey, respondents were invited to provide contact information for future research. In Fall 2020, the team recontacted consented participants for the Trans PULSE Canada COVID Study. The 1187 individuals who had consented to recontact were sent invitations via their preferred contact mode(s) (email, text message, telephone), in the language in which they originally participated. Participants had the option to complete the survey via a paper copy, but all questionnaires were ultimately completed online. The 30-minute-long questionnaire included items on demographics, social experiences, COVID-related experiences, and health status. Participants received a $20 gift card honorarium. The Trans PULSE Canada COVID Survey was approved by Research Ethics Boards at Western University, Wilfrid Laurier University, and Drexel University.

For this cross-sectional analysis, of the 820 COVID survey participants, we excluded those under 16 years of age (n = 2), as these individuals would be unlikely to facilitate their own care outside of parental guidance, as well as those who did not have a primary care provider (n = 126) or had missing primary care provider data (n = 3). The final analytic sample size was n = 689.

### 2.2 Measures

Self-rated mental health, the exposure of interest, was measured using a question from Statistics Canada’s Canadian Community Health Survey (Statistics Canada, 2018): “In general, would you say your mental health is…?” with response options including “Excellent,” “Very Good,” “Good,” “Fair,” and “Poor.” Responses were dichotomized as either “Fair or Poor” or “Good to Excellent.” As a validated measure for population mental health research, self-rated mental health is highly correlated with clinical multi-item measures of psychological distress and mental health service use (Ahmad et al., 2014). Participants were asked if they had a regular primary care provider (family doctor or nurse practitioner); those who had a primary care provider were asked: “Since March 12, 2020 have you avoided talking to your primary care provider about any health concerns?” Response options included “Yes,” “No,” “I have not needed primary care during this time,” and “My primary care provider is not seeing non-urgent patients during this time.” For regression analyses, responses were dichotomized as avoidance of care (“yes”) vs. no avoidance of care (all other options). Those answering “Yes” were then asked, “What were your reasons for avoiding primary care since March 12, 2020?” with options including “Concern about being exposed to COVID-19,” “My health concern didn’t seem urgent or important enough,” “Couldn’t bring a support person to appointments,” “Couldn’t access patient navigator,” “Concern about how my voice would be perceived over the phone,” and “Other, please specify” for reasons that did not fit into the pre-specified options. Virtual care avoidance was assessed through the question “Since March 12, 2020 have you avoided accessing virtual or tele healthcare because you’re trans or non-binary?” with response options being “Yes” or “No.”.

Covariates were selected based on identification of a sufficient set of confounders using a directed acyclic graph, and included age (nine groups from 16-19 to 65+), ethno-racial background (Indigenous, non-Indigenous racialized, white), gender identity (man or boy, woman or girl, Indigenous or cultural gender, non-binary), education level (≤high school; some college, university, or Collège d’enseignement general et professionnel (CEGEP); college or university or professional or CEGEP degree), living in poverty (not low-income, low income, missing), current housing (stable, unstable), employment status (employed, informal economy, not employed or retired or other, student, missing), and anticipated discrimination (score from 0 to 4). All covariates were from 2020 COVID survey data, other than anticipated discrimination (only available in the 2019 survey). Household low-income status was based on Statistics Canada’s low-income measure (LIM) threshold (Statistics Canada, 2018). Anticipated discrimination was assessed using the 9-item Intersectional Discrimination Index-Anticipated, which has been evaluated for validity and reliability in a Canada/United States sample (Scheim and Bauer, 2019). Participants who answered at least 80% of items received a score.

### 2.3 Statistical analyses

Descriptive statistics were weighted using Trans PULSE Canada COVID survey weights that account for demographic differences between the baseline 2019 Trans PULSE Canada sample and the COVID survey sample to reduce bias. Bivariable and multivariable logistic regression analyses were performed to estimate odds ratios with 95% confidence intervals for the relationship between poorer self-rated mental health and primary care avoidance during the pandemic, adjusting for covariates. We conducted a pre-planned sensitivity analysis in which primary care avoidance was operationalized excluding avoidance due only to COVID-19 exposure worries (vs. no avoidance). All analyses were conducted using SAS version 9.4 (SAS Institute Inc.).

Missingness was relatively low. Of 689 participants, data were missing for household income (n = 44, 6.4%), anticipated discrimination (n = 43, 6.2%), employment (n = 15, 2.2%), age (n = 5, 0.7%), ethno-racial background (n = 4, 0.6%), gender (n = 1, 0.1%), and education (n = 1, 0.1%). For regression analyses, a category of “missing” was added to household income and employment, and mode imputation was used for age, ethno-racial background, gender, and education. For the anticipated discrimination score, unweighted mean imputation was applied for respondents with missing values.

## 3 Results

Weighted descriptive statistics for trans and non-binary people with a primary care provider are presented in Table 1, overall and stratified by primary care avoidance. Most participants were <35 years of age (64.5%, 95% CI: 60.7, 68.3), white (80.9%, 95% CI: 77.6, 84.2), and had completed a college or university diploma or degree (57.6%, 95% CI: 53.5, 61.7). About half were employed (48.0%, 95% CI: 43.9, 52.0). A majority self-rated their mental health as fair or poor (61.2%, 95% CI: 57.2, 65.2). When asked about avoidance of care since the pandemic was declared on March 12, 2020, 25.7% (n = 185; 95% CI: 22.3, 29.2) reported that they avoided talking to their primary care provider. Of those who avoided primary care, 58.3% (95% CI: 50.7, 65.8) identified as non-binary, versus 43.5% (95% CI: 38.8, 48.3) of those who did not avoid care.

**Table 1:** Characteristics of Trans PULSE Canada COVID Cohort Participants with a Primary Care Provider.

Total (N = 689)			Avoided Primary Care (N = 185)		Did not Avoid Primary Care (N = 504)	
Variable	N	Weighted % (95% CI)	N	Weighted % (95% CI)	N	Weighted % (95% CI)
**Age**						
16–19	39	7.5 (5.1, 9.9)	14	10.9 (5.2, 16.7)	25	6.3 (3.8, 8.9)
20–24	111	17.7 (14.5, 20.9)	34	19.5 (13.4, 25.6)	77	17.1 (13.4, 20.8)
25–29	141	21.6 (18.2, 25.0)	42	24.5 (17.8, 31.2)	99	20.6 (16.7, 24.5)
30–34	123	17.6 (14.6, 20.7)	34	16.8 (11.3, 22.3)	89	17.9 (14.3, 21.5)
35–39	101	12.6 (10.1, 15.1)	22	10.4 (6.0, 14.9)	79	13.3 (10.3, 16.4)
40–44	59	7.1 (5.1, 9.0)	16	7.3 (3.7, 11.0)	43	7.0 (4.7, 9.2)
45–49	49	5.9 (4.1, 7.7)	12	4.8 (1.9, 7.8)	37	6.3 (4.1, 8.5)
50–54	20	3.0 (1.6, 4.4)	3	1.6 (0.0, 3.4)	17	3.5 (1.7, 5.2)
65+	46	7.0 (4.9, 9.1)	8	4.1 (1.2, 6.9)	38	8.0 (5.4, 10.6)
**Ethno-racial background**						
Indigenous	50	9.0 (6.4, 11.5)	19	13.1 (7.4, 18.9)	22	7.5 (4.8, 10.3)
Non-Indigenous racialized	73	10.1 (7.7, 12.5)	18	8.7 (4.6, 12.8)	64	10.6 (7.7, 13.5)
White	566	80.9 (77.6, 84.2)	148	78.1 (71.5, 84.7)	418	81.9 (78.1, 85.6)
**Gender Identity**						
Man or boy	170	25.3 (21.7, 28.9)	37	18.9 (13.0, 24.8)	133	27.5 (23.2, 31.8)
Woman or girl	173	24.7 (21.2, 28.2)	37	19.0 (13.1, 24.9)	136	26.7 (22.5, 30.9)
Indigenous or cultural identity	18	2.6 (1.4, 3.9)	6	3.7 (0.7, 6.8)	12	2.3 (0.9, 3.6)
Non-binary	328	47.3 (43.3, 51.4)	105	58.3 (50.7, 65.8)	223	43.5 (38.8, 48.3)
**Education Level**						
High school or less	80	16.1 (12.7, 19.4)	18	13.8 (7.6, 20.0)	62	16.8 (12.9, 20.8)
Some college, university, CEGEP^b^	176	26.3 (22.7, 29.9)	63	34.8 (27.5, 42.1)	113	23.4 (19.3, 27.5)
College, university, CEGEP^b^ degree	433	57.6 (53.5, 61.7)	104	51.4 (43.7, 59.1)	329	59.8 (55.0, 64.6)
**Living in Poverty**						
Not low-income	390	55.0 (50.9, 59.0)	88	46.9 (39.2, 54.5)	302	57.8 (53.0, 62.5)
Low-income	255	37.5 (33.6, 41.5)	85	45.6 (37.9, 53.2)	170	34.7 (30.2, 39.3)
Missing	44	7.5 (5.2, 9.8)	12	7.6 (3.2, 12.0)	32	7.5 (4.8, 10.2)
**Current Housing**						
Stable	648	93.3 (91.2, 95.4)	176	94.1 (90.2, 98.0)	472	93.0 (90.5, 95.5)
Unstable	41	6.7 (4.6, 8.8)	9	5.9 (2.0, 9.8)	32	7.0 (4.5, 9.5)
**Employment Status**						
Employed	343	48.0 (43.9, 52.0)	86	44.3 (36.7, 52.0)	257	49.2 (44.5, 54.0)
Informal economy	14	1.8 (0.9, 2.8)	2	1.0 (0.0, 2.5)	12	2.1 (0.9, 3.3)
Not employed, retired, other	164	23.7 (20.3, 27.2)	50	26.3 (19.6, 33.1)	114	22.8 (18.8, 26.8)
Student	153	24.1 (20.6, 27.7)	45	27.0 (19.8, 34.1)	108	23.1 (19.0, 27.3)
Missing	15	2.3 (1.1, 3.6)	2	1.3 (0.0, 3.2)	13	2.7 (1.1, 4.3)
**Anticipated Discrimination Score (mean (95% CI))**						
	646	2.4 (2.3, 2.4)	175	2.6 (2.5, 2.8)	471	2.3 (2.2, 2.4)
Missing	43	–	–	–	–	–
**Self-Rated Mental Health**						
Excellent-good	256	38.8 (34.8, 42.8)	41	21.7 (15.5, 28.0)	215	44.7 (40.0, 49.5)
Fair-poor	433	61.2 (57.2, 65.2)	144	78.3 (72.0, 84.5)	289	55.3 (50.5, 60.0)
**Avoidance of PCP**^a^						
No	504	74.3 (70.8, 77.7)	–	–	–	–
Yes	185	25.7 (22.3, 29.2)	–	–	–	–
**Reasons for PCP**^a^**Avoidance**						
Concern about COVID exposure	–	–	46	26.5 (19.5, 33.4)	–	–
Non-urgent/important health concern	–	–	134	72.7 (65.9,79.5)	–	–
Support person	–	–	14	7.0 (3.3, 10.7)	–	–
Patient navigator	–	–	4	2.0 (0.0, 4.0)	–	–
Concerns about voice perception	–	–	22	11.9 (6.9, 16.8)	–	–
Other	–	–	62	34.3 (26.9, 41.6)	–	–

Data are reported as N(%) unless otherwise specified. Frequencies (N) are unweighted, and proportions (%) are weighted to demographic data from the Trans PULSE Canada 2019 Survey.

a PCP = Primary Care Provider (includes family physician or nurse practitioner).

b CEGEP = Collège d’enseignement général et professionnel.

Among those who had avoided care, 72.7% (n = 134; 95% CI: 65.9, 79.5) indicated that one reason for avoiding (participants could select more than one) was that they perceived their health concern as non-urgent or unimportant. Write-in responses in the “other, please specify” category comprised the second largest (34.3%, 95% CI: 26.9, 41.6) proportion of the reasons for avoided care. Themes identified from write-in responses included transportation issues, mistrust or discomfort with their provider, transphobia encountered when attempting to access care, and anxiety related to scheduling an appointment or talking on the phone (for reasons other than voice perception). Forty-six participants (26.5%, 95% CI: 19.5, 33.4) reported concerns about COVID-19 exposure as a reason for avoiding care. Of those who avoided care, 21.0% (n = 36, 95% CI: 14.4, 27.5) also reported avoidance of virtual healthcare.

The results of logistic regression models predicting avoidance of care based on fair/poor self-rated mental health are presented in Table 2. After controlling for confounders, those with worse mental health were more likely to avoid primary care than those with better mental health (adjusted OR; AOR = 2.37; 95% CI: 1.50, 3.77). Results were similar in the sensitivity analysis (not shown) examining the association between self-rated mental health and avoidance of primary care for non-COVID reasons (AOR = 2.52; 95% CI: 1.52, 4.17; n = 643).

**Table 2:** Association between fair-poor mental health and primary care avoidance among Trans PULSE Canada COVID Cohort participants (n = 689).

	Unadjusted odds ratio (95% CI)	Adjusted odds ratio^a^ (95% CI)
Good to excellent mental health	1.00	1.00
Fair or poor mental health	2.91 (1.92, 4.42)	2.37 (1.50, 3.77)

a Adjusted for age, education level, employment status, ethno-racial background, gender identity, current housing, household income, and anticipated discrimination.

## 4 Discussion

In this community-based sample of trans and non-binary people in Canada, there was a high prevalence of primary care avoidance (25.7%) and fair or poor self-rated mental health (61.2%) during the COVID-19 pandemic. After controlling for potential confounders, participants with worse mental health were more likely to avoid primary care during the pandemic. This is concerning as postponing care may increase the risk of morbidity and mortality (Czeisler et al., 2020).

Within the existing literature, the prevalence of healthcare avoidance due to anticipated discrimination among trans and non-binary people has ranged from 16.1% over a 4-month period during the pandemic to 22.8% over a 12-month period in the 2015 U.S. Trans Survey (Kcomt et al., 2020, Mason, 2021). While our questionnaire asked about avoidance of care within a broader context, our results nonetheless demonstrated a comparable prevalence. Among the reported reasons for primary care avoidance, the most common was that the respondent did not consider their health concern urgent or important enough. The overwhelming focus on COVID-19 testing and care within the Canadian healthcare system between March and September 2020, paired with service reductions, could have made some participants feel that their non-COVID-related healthcare needs were relatively less important. Although approximately a quarter of respondents who avoided care (26.5%) indicated fear of COVID-19 exposure as a reason for avoidance, the relationship between self-rated mental health and avoidance was similar when excluding those who reported this reason. This suggests that the relationship between poorer mental health and primary care avoidance was not driven solely by anxiety related to COVID-19. Nevertheless, these may be connected. For participants with poorer mental health and consequent reduced functioning, the threshold to deem health concerns urgent, or to access care for non-urgent concerns, or to deal with misgendering or attending healthcare visits alone, may have been higher than among those with better mental health, particularly in the context of heightened COVID risk. Additionally, unlike in the United States where lack of health insurance coverage may limit access to care for trans and non-binary populations (Jarrett et al., 2020), financial barriers would not have played as large of a role in avoidance for participants in this Canadian study.

Themes within the open-response category for reasons for avoidance raised structural and operational issues including transportation problems, a lack of trust and a low level of comfort with the provider, transphobia encountered when attempting to access care, anxiety about a pressing health issue, and high levels of anxiety, depression, or distraction preventing the arrangement of an appointment or talking on the phone. Some of these responses point to interpersonal and structural stigma that compromise access to healthcare for trans and non-binary individuals, and that led to healthcare avoidance prior to the pandemic (White Hughto et al., 2015, Bauer et al., 2009).

As our analyses controlled for anticipated discrimination, however, the results indicate an association between mental health status and primary healthcare avoidance that is independent of anticipated discrimination. Amidst a pandemic with isolation causing decreased access to support persons and mental health services (Zhou et al., 2020), mental health and care navigation supports for trans and non-binary populations should be prioritized.

### 4.1 Implications for clinical practice and medical education

To improve both physical and mental health outcomes, we suggest increasing access to care through virtual visits and other methods of remote communication. Online forums, videoconferences, text-messaging, smartphone apps, and email are all useful communication strategies for health service delivery (Zhou et al., 2020). Virtual connections could serve to bypass transportation issues and avoid misgendering from clinic staff. When used with an interprofessional approach and with providers that have gender-affirming care experience, telehealth has the potential to help reduce anxiety among these patient populations (Ng et al., 2021). However, 21.0% of participants who avoided primary care in this study also reported avoiding virtual care. As greater emphasis is placed on telehealth and virtual care services in response to COVID-19 (Ole-Petter et al., 2020), further research is needed to understand barriers and facilitators to virtual care for trans and non-binary people. Multimodal care options should be maintained as preferences for virtual versus in-person care may differ between patients.

To reduce future care avoidance, proactive assessment of mental health symptoms and referrals to gender-affirming mental health providers may be beneficial. Trans and non-binary community resources can also be leveraged to support mental health and facilitate care navigation. For instance, among social work clinicians who offer mental health services in Canada, gender affirming therapy has been adapted to be delivered through telehealth platforms, and these services can help build social support networks as resources available outside of scheduled appointments (Craig et al., 2021).

While providers are responsible for delivering equitable care and have the power to increase some aspects of healthcare accessibility for trans and non-binary patients, a multi-level approach is needed. Promotion of health equity ought to occur within medical training institutions and primary care practices, and in addressing structural barriers within the larger health systems in which providers practice. Given the paucity of medical schools and residency training programs that provide sufficient education on how to create a gender-affirming healthcare environment (Obedin-Maliver et al., 2011, Ufomata et al., 2020), our results highlight the need for greater inclusion of trans and non-binary health topics in medical education (Schreiber et al., 2021). Existing literature suggests a longitudinal curriculum be implemented with an emphasis on clinical skills (Dubin et al., 2018, Park and Safer, 2018, McDowell et al., 2020). Practicing clinicians also have opportunities to foster affirming environments by providing personalized care to their patients through continuing education, self-reflection, and practice to reduce implicit bias (McDowell et al., 2020). Structurally, healthcare systems can tackle stigma through gender identity and expression anti-discrimination policies, through the employment of more gender minority individuals, and through staff training in gender-affirming care (McDowell et al., 2020; McClain et al., 2016).

Such initiatives must consider gender, ethnoracial, and other forms of diversity within trans and non-binary populations. As our sample was predominantly white, we lacked statistical power to investigate how experiences of primary care avoidance during the pandemic may have varied for trans and non-binary Indigenous and/or racialized individuals. Previous research, however, has demonstrated a higher likelihood of avoidance of care due to anticipated discrimination for racialized trans people (Kcomt et al., 2020). Additionally, transfeminine people in Ontario, Canada who were Indigenous and/or racialized were less likely to have a regular family provider than other trans people (Scheim et al., 2017). Healthcare providers and systems can address the particular healthcare barriers faced by trans and non-binary people of color by being aware of both transphobic and racist microaggressions and developing strategies that help to reduce their implicit bias. They can also actively assess and support patient strengths and resilience in navigating multiple oppressions (Chang and Singh, 2016).

### 4.2 Study limitations

Some limitations of the study should be noted. Although we applied survey weights such that these data represent the demographics of the full 2019 Trans PULSE Canada survey (n = 2873), the baseline survey used convenience sampling, with recruitment through trans community events and networks. Those who were less connected to trans communities may have been less likely to participate, and results cannot be generalized to all trans and non-binary people in Canada.

With the cross-sectional nature of this study, causality between poor mental health and primary care avoidance cannot be inferred. It is possible that those who avoided primary care also received fewer mental health services from their providers, and subsequently experienced worse mental health. Finally, participants who responded to the question about primary care avoidance by indicating “my primary care provider is not seeing non-urgent patients during this time” were not classified as avoiding care, but we did not collect data on whether they had actually attempted to access care. Given that the most common reason for self-reported avoidance was the perception that one’s health issues were non-urgent, it is possible that some of these respondents were indeed avoiding care. As a result, the prevalence of primary care avoidance may be underestimated.

## 5 Conclusion

In a sample of trans and non-binary people in Canada with a primary care provider, we found that about one-quarter reported avoiding care during the first half-year of the COVID-19 pandemic, and poorer mental health was associated with increased odds of avoidance. These results highlight the importance of addressing mental health needs among trans and non-binary individuals living through the COVID-19 pandemic and beyond. Amidst the pandemic, healthcare providers should consider offering multiple modes of care to trans and non-binary patients, including virtual methods of communication for referral and delivery of physical and mental health services. Finally, it is paramount that healthcare providers in training receive education specific to trans and non-binary health, and that clinicians in practice take appropriate steps to reduce stigma and increase the accessibility of their services for trans and non-binary patients.

## CRediT authorship contribution statement

**Abigail Tami:** Conceptualization, Methodology, Formal analysis, Software, Writing – original draft. **Tatiana Ferguson:** Conceptualization, Investigation, Resources, Writing – review & editing, Funding acquisition. **Greta R. Bauer:** Methodology, Data curation, Writing – review & editing, Funding acquisition. **Ayden I. Scheim:** Conceptualization, Methodology, Supervision, Writing – review & editing, Project administration, Funding acquisition.

## Declaration of Competing Interest

The authors declare that they have no known competing financial interests or personal relationships that could have appeared to influence the work reported in this paper.
